# T-Time: A data repository of T cell and calcium release-activated calcium channel activation imagery

**DOI:** 10.1186/s13104-017-2739-x

**Published:** 2017-08-15

**Authors:** Cody Arbuckle, Milton Greenberg, Adrienne Bergh, Rene German, Nick Sirago, Erik Linstead

**Affiliations:** 1 Schmid College of Science and Technology, Chapman University, One University Drive, Orange, CA 92866 USA; 2 Department of Physiology and Biophysics, University of California, Irvine, CA 92697 USA; 3 Anivive Life Sciences Incorporated, Long Beach, CA 90808 USA

**Keywords:** T cell, Phase contrast imagery, Live-cell dynamics

## Abstract

**Background:**

A fundamental understanding of live-cell dynamics is necessary in order to advance scientific techniques and personalized medicine. For this understanding to be possible, image processing techniques, probes, tracking algorithms and many other methodologies must be improved. Currently there are no large open-source datasets containing live-cell imaging to act as a standard for the community. As a result, researchers cannot evaluate their methodologies on an independent benchmark or leverage such a dataset to formulate scientific questions.

**Findings:**

Here we present T-Time, the largest free and publicly available data set of T cell phase contrast imagery designed with the intention of furthering live-cell dynamics research. T-Time consists of over 40 GB of imagery data, and includes annotations derived from these images using a custom T cell identification and tracking algorithm. The data set contains 71 time-lapse sequences containing T cell movement and calcium release activated calcium channel activation, along with 50 time-lapse sequences of T cell activation and T reg interactions. The database includes a user-friendly web interface, summary information on the time-lapse images, and a mechanism for users to download tailored image datasets for their own research. T-Time is freely available on the web at http://ttime.mlatlab.org.

**Conclusions:**

T-Time is a novel data set of T cell images and associated metadata. It allows users to study T cell interaction and activation.

## Background

Live-cell imaging allows for the in-depth study of activities occurring within living cells. The understanding of these complex interactions has wide ranging implications to many biomedical applications [1]. As the techniques used in live-cell imaging have improved, they have become an important component of monitoring the interactions within and among cells throughout their lifecycle [2, 3] . Several probes have been developed in relation to these endeavors, [4–6], and in turn, we have created T-Time, a repository of T cell images using a novel tag developed in [7].

T cells are lymphocytes that play a central role in cell-mediated immunity to foreign pathogens. Dysregulated T cell responses are implicated in numerous chronic conditions ranging from severe combined immunodeficiency (SCID) to autoimmunity and cancer. T cells migrate extensively throughout the body to enable proper immune function. This migration is the focus of extensive research and has motivated the development of immunosuppressive drugs which specifically inhibit T cell migration [8]. Despite the ongoing clinical applications of the suppression of T cell crawling, the molecular mechanisms that facilitate it are incompletely characterized. The activity of Ca$^{2+}$ channels is required for basal T cell motility [9] and increases in internal Ca$^{2+}$ concentrations have been previously shown to suppress T cell migration, facilitating sustained interactions between T cells and other leukocytes [10]. Characterization of subcellular Ca$^{2+}$ dynamics in migrating T cells has been limited due to a lack of sensitive probes that enable rapid ratiometric quantification of internal Ca$^{2+}$ concentrations.

Ca$^{2+}$ diffuses rapidly through the cytoplasm of activated cells, but rapid imaging techniques have described local Ca$^{2+}$ microdomains at the immunological synapse [11]. Ca$^{2+}$ microdomains have been previously shown to refill peripheral endoplasmic reticulum Ca$^{2+}$ stores juxtaposed to the plasma membrane and to regulate plasma membrane-restricted enzymes including the Ca$^{2+}$ ATPase pump and adenylyl cyclase. Nonetheless, little is known about the composition or function of Ca$^{2+}$ microdomains or how they facilitate T cell migration. Ca$^{2+}$ oscillations are essential for T cell stopping behavior and Ca$^{2+}$ signaling through the Ca$^{2+}$ release-activated Ca$^{2+}$ (CRAC) channel has recently been shown to enable long-range T cell migration between organs [7]. In order to understand the effect Ca$^{2+}$ microdomains and CRAC channel components have on T cell crawling, it is desirable to explore models that are capable of capturing T cell migration as well as local Ca$^{2+}$ concentrations and the location of CRAC channel proteins within the T cell. To this end, T-Time provides a repository of data which allows for tracking and identifying T cells and CRAC channels over an extended period of time, and can be used to build and tune such models.

**Fig. 1:**
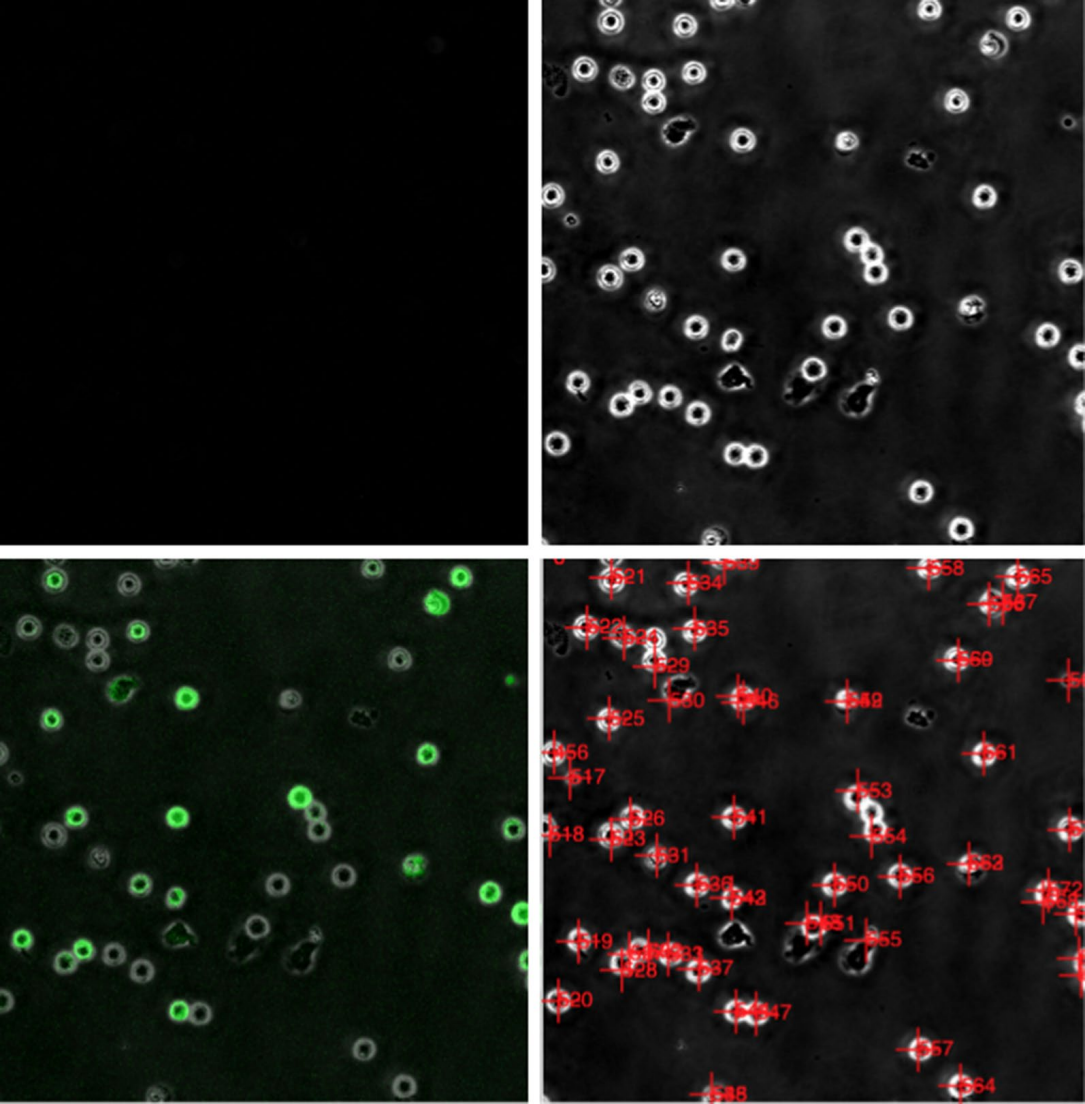
Image phase comparison. *Top left*: unenhanced image,* top right*: enhanced image, *bottom left*: enhanced image with phase overlay,* bottom right*: enhanced image with tracking

In addition to information regarding the relationship of CRAC channel activation and T cell motility, it is also useful to understand the effect that CRAC channels have on the interaction between regulatory T cells and T cells. Regulatory T cells are a subpopulation of T cells which have been shown to suppress or down regulate induction and proliferation of effector T cells [12]. Regulatory T cells are notoriously hard to study due to their expression of CD4 and CD25, which are also expressed by effector T cells, making the distinction between regulatory T cells and effector T cells difficult. By leveraging new probes the data housed in T-Time allows the interaction between these cells and the resulting impact of CRAC channel activation to be more easily studied by the scientific community using a standard, independent data repository. An example set of the images available can be seen in Fig. 1.

In this paper we present the largest publicly available repository of time-lapse sequences containing the motility data of several 100 T cells along with 50 time-lapse sequences containing images of the interaction between regulatory T cells and T-cells. Accompanying metadata includes location, morphological changes, and movement of T cells throughout the time-lapse, as well as the activation of the CRAC channel. Additionally, in the motility datasets the movement of each individual T-cell throughout the time lapse has been marked and tracked for its time in frame, resulting in several thousand T cell tracks and their accompanying CRAC channel activation.

T-Time is available at http://ttime.mlatlab.org. End users may download imagery with varying degrees of processing, ranging from raw, unenhanced images to enhanced, tracked cells. Moreover, metadata files containing the location of individual cells, and in-depth views of their summary statistics are provided. For computational researchers, the database is a source of raw data and processed measures that can be used to develop new algorithms for tracking, segmentation, and bioinformatic approaches to link signaling molecule activation with overall cell functionality. For biologists, the visualizations and summary statistics allow for further understanding of T cell movement, cell-to-cell interactions, and CRAC channel activation that may be further utilized to test therapeutics targeting inflammatory disorders.

## Methods

### Data collection

Human peripheral blood leukocytes were isolated from blood of voluntary healthy donors by Ficoll-Hypaque (gradient = 1.077 g/dl) density gradient centrifugation. Human CD3+ T cells were isolated using EasySep Human CD3+ Isolation kits from StemCell Technologies according to manufacturer’s instructions. The purity of CD3+ cells was confirmed to be greater than 95% by flow cytometry.

Primary human T cells were transfected by nucleofection with GCamp6f DNA, using the manufacturer’s protocol (Amaxa, Allendale, NJ). GCaMP, a genetically encoded calcium indicator (GECI) is generated from a combination of calmodulin, green fluorescent protein and M13, a peptide sequence from myosin light chain kinase. There is an induction of a change in fluorescence signal, upon binding of GECI to Ca$^{2+}$, allowing for the measurement of receptor activation events, which trigger Ca$^{2+}$ fluxes. Briefly, T cells were centrifuged at 200×*g* for 10 min and supernatant was completely aspirated. T cells were resuspended in Amaxa nucleofector solution with 1 μg of DNA. T cells were transferred into a Nucleocuvette™, a cuvette coated with a conductive polymer electrode, suitable for electroporation. The cuvette was inserted into a Nucleofector™ 2b Device, and transfected using the high-viability protocol.

Human T cells were maintained in human T cell culture media containing RMPI 1640 supplemented with 10% fetal bovine serum. Following transfection, T cells were immediately transferred via pipette into a 12-well plate containing T cell culture media pre-warmed to 37 °C. Cell culture plates were maintained in a 37 °C humidified incubator at 5% CO_2_ to ensure viability. Human cells were used for experiments 24 hours after transfection.

Transfected T cells were activated overnight prior to T-Treg imaging. To achieve this, 12 well plates were coated with a 10 μg/ml solution of anti-CD3 in sterile PBS for 2 h at 37 °C. The antibody solution was then aseptically decanted from the microwell plate and washed three times with sterile PBS. Freshly transfected T cells were then plated in complete human T cell media, as described previously.

Reagents used in data collection were from the following sources:

- Ficoll Blood Cell Purification: Sigma-Aldrich Histopaque-1077, Catalog Number: 10771.
- T Cell Enrichment: StemCell Technologies EasySep Human T Cell Enrichment Kit, Catalog Number: 19051.
- Human T Cell Transfection kit: Lonza Amaxa Human T Cell Nucleofector Kit, Catalog Number: VPA-1002.
- Human T Cell Culture Medium: ThermoFischer RMPI 1640 Medium, Catalog Number: 11875093.
- Fetal Bovine Serum: ThermoFischer OneShot format, Catalog Number: A3160401.
- Imaging Dish: MatTek Corp. 35 mm Dish, No 1.5 Coverslip, Catalog Number: P35G-1.5-14-C.
- ICAM-1: R&D Systems Recombinant Human ICAM-1/CD54 Fc Chimera, Catalog Number: 720-IC.
- Bovine Serum Albumin: Sigma-Aldrich Bovine Serum Albumin, Lyophilized Powder, Catalog Number: A9418.
- Phosphate Buffered Saline: ThermoFischer Phosphate Buffered Saline, Catalog Number: 10010001.
- Treg Cell Enrichment: StemCell Technologies EasySep™ Human CD4+CD25+ T Cell Isolation Kit, Catalog Number: 18062.
- T Cell Activating Antibody: BioLegend LEAF™ Purified anti-human CD3 Antibody, Catalog Number: 317303.

For in vitro T cell migration video microscopy, 35-mm glass bottom microwell dishes were coated overnight with 3 μg/ml intercellular adhesion molecule-1 (ICAM-1)-Fc, and then blocked with 2% bovine serum albumin (BSA) in phosphate buffered saline (PBS). Transfected T cells were added and allowed to settle for 30 min at 37 °C, and non-attached cells were removed by gentle washing. No deliberate activation was needed for the migration protocol as the green flashes detected in imaging are spontaneous florescence that occur during migration as a result of the transfection with GCamp6f. Images of migration were taken at 37 °C using an Olympus FluoView FV10i microscope.

Following overnight culture, imaging chambers suitable for visualizing T cell-Treg interactions were prepared. MatTek imaging dishes were coated with a 0.1 mg/ml solution of poly-l-lysine in sterile PBS for 2 h at 37 °C. Poly-l-lysine facilitates the attachment of T cells to the cover glass, preventing cellular migration. Tregs were then freshly isolated according to the manufacturer’s protocol and co-cultured with activated T cells in the imaging dish. Cells were allowed to adhere to the cover glass by incubation for 1 h at 37 °C in the Olympus FluoView FV10i microscope. To activate the CRAC channel, a 10 μg/ml solution of anti-CD3 was added to the imaging dish, and time-lapse microscopy was initiated immediately.

### The T-Time repository

T-Time is freely available on the web at http://ttime.mlatlab.org, hosted by the machine learning and assistive technology (MLAT) Lab at Chapman University. The T-Time data repository is implemented with the open-source relational database MySQL (v. 5.7.11) [13], and the accompanying web interfaces are developed using the Laravel PHP Framework [14] and jQuery [15]. The image enhancement and tracking algorithms for processing the raw data were developed using Matlab (v.2015b) and its image processing, bioinformatics, and statistics packages. The code for image enhancement and tracking is actively maintained and available for download from the T-Time site. T-Time is deployed on a 12-core server containing 256 GB of physical memory. To increase performance the data is stored on redundant 256 GB solid-state drives (SSDs). The database and web interface have been tested and verified to support multiple concurrent users.

In the sections that follow, we describe the key components of T-Time in detail.

#### Data storage

The use of a normalized database structure, along with the storage of the images on a local SSD, allow for fast and efficient querying and access to the processed images. The usage of such a schema enables investigation of cells at similar time points in a similar environment which aids in the creation of an accurate model of cellular dynamics. Furthermore, this schema will allow for new image data and types to be quickly added to T-Time in the future.

To populate the T-Time database, processing began with raw data in Tagged Image Format (.tif) and proceeded through a series of steps to enhance imagery, identify cells of interest, and track them over time. These steps are described in detail in the following section.

The set of 71 time-lapse sequences containing T cell movement and CRAC channel activation had an average of 160 time points with 3 phases per time point; the average unprocessed .tif file had a size of 525 KB. Further processing of each time point resulted in a total of 10 images, 5 enhanced and untracked along with 5 enhanced and tracked. All enhanced and untracked files had an average size of 550 KB, while the tracked images had an average size of 725 KB.

The set of 50 time-lapse sequences of T cell activation and T reg interactions had an average of 70 time points with 1 phase per time point, and the average unprocessed .tif file had a size of 525 KB. Following processing, each time point yielded in 2 images, 1 enhanced and untracked as well as 1 enhanced and tracked image. The tracked images had an average size of 1.2 MB while the untracked images had an average size of 525 KB.

These processing steps resulted in a combined 40 GB of data that comprises the T-Time repository.

#### T-Time image processing

Following the capture of images, each image was imported into Matlab. The intensity values in the image were mapped and adjusted in such a manner that 1% of the data became saturated at low and high intensities of the original image. This method further increased the contrast change allowing for more accurate cell detection. Due to the circular geometry of the majority of cells, as well as a desire to increase the efficiency of the algorithm, a function designed specifically to capture circular objects was implemented to allow for the separation of easily distinguishable cells. In order to detect clumped cells and compensate for the varying illumination levels obtained in live-cell imaging, each slide was subsequently divided into sub-images of 32 × 32 pixels, which were slightly larger than the experimentally determined average diameter of the cells in question.

**Fig. 2:**
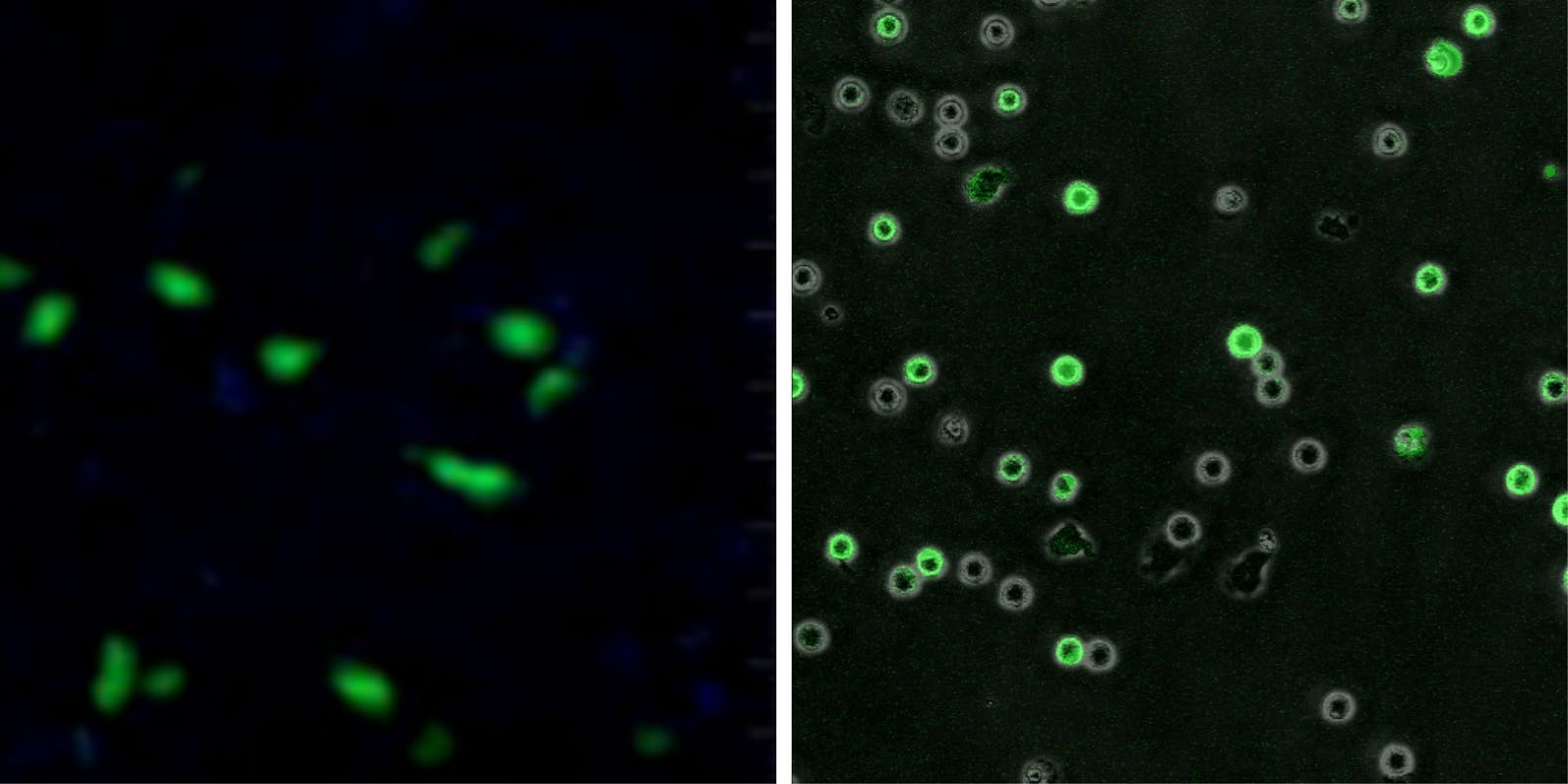
Image enhancement method comparison. Figure comparing 2 phase contrast images. One enhanced with the commercially available Imaris software (*right*) and the other with the recently released T-Time image enhancement algorithm (*left*)

A thresholding technique was next applied to each gray-scale image to increase the contrast, resulting in a higher quality image than available with contemporary software as seen in Fig. 2. The constraints of the thresholding values were set to experimentally determined parameters, prohibiting those sub-images that did not contain cells from contributing a significant amount of noise to the image as a whole. Once a threshold had been established for a sub-image, it was converted to a binary sub-image using the specific threshold determined for it. A function capturing specific parameters for each connected component in the sub-image was then applied to each binary sub-array in order to identify the cells that were contained within.

Following the collection of all edges and regions within each sub-image, the collected values were processed, and if the detected cells met specific parameters, their centroid location, area, perimeter, minor and major axis length, and maximum pixel intensity were collected and stored. In order to ensure that cells which fell on the boundary of a sub-image were not ignored or counted twice, the edges of each sub-image were processed to see if cells of interest existed along these edge spaces. If cells were determined to be near each other and on the edge of a sub-image, a new image was created around this area of interest and all cells detected within this area of interest on the first processing phase were replaced with the ones detected in the secondary processing phase. Repeating the same process with a shifted focal point ensured that only unique cells were detected.

Upon completion of the preliminary analysis of a slide, those cells that were detected and deemed unimportant by the domain expert, such as those showing no calcium activity, or those which were not T-cells, were discarded. The removal of these cells was accomplished by matching the characteristics given by the domain expert to the data collected from each image. A copy of the original image was made and the pixel values of the “noise” cells were reset to a background pixel value to effectively remove them from the image.

**Fig. 3:**
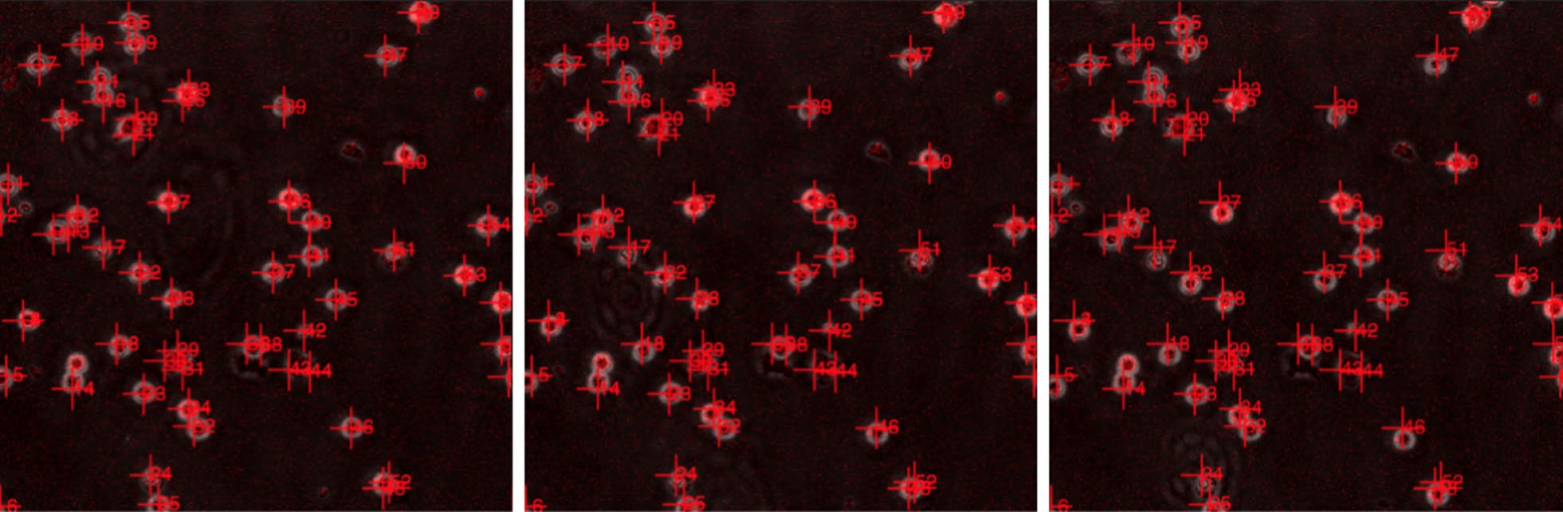
Tracked images. Three successive time-lapse images displaying the tracking and enhanced images available in the database. Additionally the presence of several 100 cells over just three time points highlights the need for an automated tracking methodology

Following the successful identification of all cells, in all slides, an additional processing step was to determine the movement of each cell individually. The parameters of each cell, at each time point, were compared to the information collected from near-by cells in subsequent time points. By matching the characteristics of cells that were found in close proximity in subsequent frames, each cell could be successfully tracked throughout its time in the field of view, an example of the cell tracking results can be seen in Fig. 3. Using this approach allowed the algorithm to cope with cells that stayed stationary for long periods of time, cells that entered the field of view after the initial slide and cells that left the field of view before the completion of the time series.

**Fig. 4:**
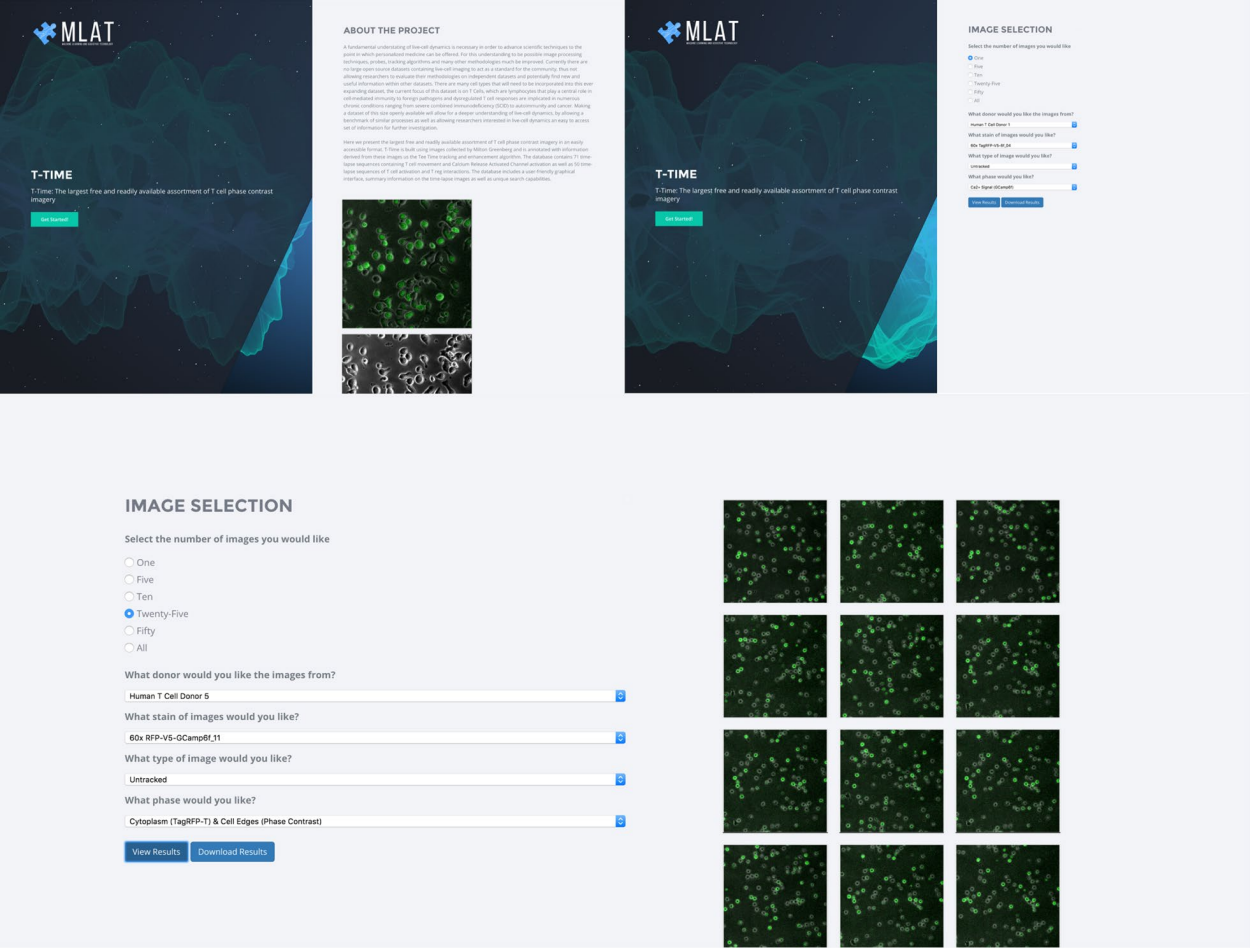
T-Time User Interface. A composite display of pages on ttime.mlat.org. (Clockwise starting from *top left*). The homepage for the site, containing information about the project. Upon clicking the “Get Started” button the user is directed to screen-shot two. Screen-shot two depicts the image selection options, allowing a user to select from the previously mentioned options. Upon completing a selection the user can directly download all images and summary data as a .zip file using the “Download Results” button or view the results in a separate page using the “View Results” button. Selecting “View Results” will direct the user to Screen-shot three which shows displays the selected images along with the search page along with the same search functionality as seen on the previous page. The option to upload images for processing be added to the homepage for the site as well as the option to predict the movement of a cells given a specific set of factors such as cell population, field size and cell type

#### Web interface

Figure 4 depicts a composite screen shot from the T-Time web interface, demonstrating a search selecting a specific date, strain type, image type, and tracking type. A researcher interested in testing the effectiveness of a newly developed tracking algorithm can select a full time sequence of enhanced images without tracking. All images will then be displayed on the site, and can be downloaded to a .zip archive. Additionally, a file containing summary information about each detected cell (such as the mean pixel intensity, max pixel intensity, major axis length, minor axis length, area, and circumference) is included in the .zip file. The researcher can use the contents of these files to train their new tracking algorithm. In order to further validate the new tracking algorithm the user can download the tracked images and compare the results to the previously tracked cells. By downloading the tracked cell dataset the user gains access to the metadata which also includes positional information for each cell over time. Additionally, the usage of the tracked images allows for a researcher to speed up the feature detection necessary to create a classification or detection algorithm for T cells. For researchers interested in creating methodologies for automatically classifying a large number of cell types, the images provided by the T-Time database provide a large number of identified T cells which can serve as training data.

For biologists interested in examining cell motility, or T cell interactions, the researcher can select tracked images, allowing them to easily distinguish cells from background noise in the images. Additionally the image enhancement techniques used allow for sharper image boundaries and clearer differentiation between tightly clustered cells. A researcher interested in the effects of CRAC channel activation on the shape and motility of a cell can download the image overlays, allowing them to see CRAC channel activity in relation to the entire cell.

## Results

### T cell movement and CRAC channel activation statistics

Table 1 contains the summary statistics of the T cell movement and CRAC channel activation dataset, calculated using the T-Time database and T-Time image and enhancement algorithm. All measurements were calculated in pixels in order to allow for generalization of the algorithm over different image capture methods. Throughout all datasets the average area of each T cell was calculated at 242.86 pixels with a standard deviation of 113.07 while the perimeter of each cell was calculated by measuring the total length of plasma membrane exposed, and had a mean value of 82.90 with a standard deviation of 30.23. The standard deviation values can be explained by the shape shift that occurs as cells morph from spherical to rod-like forms as a result of CRAC channel activation. This shape change is detected through the measurement of major and minor axes, given by the longest and shortest distances possible across each cell. The distance measurement is calculated by determining the distance between the centroids of a given tracked cell pictured at two subsequent time points. This measure shows that there is a wide range in the movements the cells experience throughout the time series; some cells remain inactive throughout the entire time-series while others are highly mobile. The extent measure is the ratio of pixels in the region to the pixels in the bounding box, which provides a way to ensure that the bounding box used to track cells is not too large. The average value is 0.60 and the max value is 0.91 proving that all cells were captured only once, since no bounding boxes were completely filled. The max intensity value is a measure of the highest pixel value contained within a cell, typically showing the degree of CRAC channel activation. Throughout all time series, the majority of cells generally have at least some activation which explains the high mean value for max intensity. The mean intensity value is the average pixel value for the cell, which includes CRAC channel activation and can show the overall activation of CRAC channels throughout the entire cell.

**Table 1:** Summary statistics for T cell movement and CRAC channel activation dataset (all measurement are in pixels with a resolution of 61305 pixels/inch)

Measure	Min	Mean	Max	Standard deviation
Area	75.00	242.86	450.00	113.07
Perimeter	31.79	82.90	195.15	30.23
Major axis	11.24	29.16	47.30	8.309
Minor axis	3.97	13.02	30.87	4.78
Distance	0.00	1.53	20.00	3.14
Extent	0.22	0.60	0.91	0.11
Max intensity	19114.00	61216.64	65535.00	12047.40
Mean intensity	17278.00	52462.48	64133.00	10724.42

### T Reg interactions statistics

Table 2 contains the summary statistics for the T Reg Interactions. Once again, all measures are calculated in pixels for generalizability. The measurements calculated from the T Reg Interactions dataset are slightly different from the T cell movement and CRAC Channel Activation because the T Reg dataset contains cells that were locked in place and thus the calculation of movement was not possible. However the lack of movement from the target cells did allow for the calculation of eccentricity, which is a measure of how far the cells are from being perfectly circular. The measure of eccentricity, combined with major and minor axis measurements, shows that these cells vary greatly in shape throughout the time series. The area and perimeter measures are calculated in the same manner as in the first dataset. However there is high variability due to the different cell types; the averages for both measures fall in the expected range but there are some outlier cells detected. The extent measure is also calculated in the same manner and reveals some outlier cells that are larger than the bounding box for this dataset. However, the shifting techniques applied to the bounding box render the error from these outliers negligible, so the T-Time cell tracking algorithm is still applied. The mean intensity value for this dataset is lower than the first dataset but this is due to different cell and stain types. The max intensity value again can be used to determine the activity of the CRAC channels throughout the time series and different levels of activity are detected.

**Table 2:** Summary statistics for T Reg interactions dataset (all measurement are in pixels with a resolution of 61305 pixels/inch)

Measure	Min	Mean	Max	Standard deviation
Area	1.00	91.79	2655.00	255.37
Perimeter	31.79	43.10	1078.40	96.77
Major axis	51.16	13.67	150.40	19.24
Minor axis	1.16	6.32	74.50	10.27
Eccentricity	0.00	0.64	0.99	0.39
Extent	0.12	0.68	1.00	0.29
Max intensity	19195.00	39010.88	65535.00	15607.79
Mean intensity	19195.00	30141.01	53124.00	6331.58

## Discussion

### Related and future works

The study of live-cell dynamics is fundamental to the development of quantitative approaches for analyzing the function of biological systems. Previous studies incorporating live-cell imaging databases have resulted in thousands of images [16, 17] which could also be applied to the study of disparate biological systems. Furthermore, there are numerous attempts to develop novel dyes and stains for use in live-cell imaging [6], yet there is little readily available data to evaluate the effectiveness of these stains on an image by image basis using established computational techniques. Along with the continued interest in live-cell imaging techniques, there has been an increased effort placed on creating computational methods to analyze this massive amount of data [18, 19], with solutions being offered by companies such as http://www.diatrack.org/ used in [20] or Imaris software used in [7]. However, there have been very few processed results released to the public for the purpose of independent evaluation the effectiveness of different practices.

Additionally, there has been wide spread adoption of large open access databases in several other scientific fields [21]. These include ChemDB, a public database containing small molecules and related chemoinformatics resources [22], Uniprot, a database of protein sequence and functional information, which was created in collaboration with several similar databases [23] and DrugBank, a database that combines detailed drug data with drug target information [24]. Using these systems as inspiration, we have provided the largest known collection of T cell phase contrast imagery to the community in order to further collaboration and aid in the establishment of novel imaging techniques.

The current T-Time repository contains only T cell imagery due to the important role that T cells play in the human body. In the future, more images containing different probes, cell types, image types and time series frequencies will be added. Creating a repository of all widely studied cells, their movements, and interactions will allow for more in-depth analysis of the factors governing cell dynamics and their role in the overall health of humans.

We are currently mining the T-Time data to study several additional T cell properties. Phenomena that are currently under investigation are changes in the thickness of the plasma membrane, the potential effect the change in cell shape has on overall motility of T cells, the mechanisms governing the cell shape change, and the overall influence the population of cells has on a single cell. The availability of this information to the community will encourage other researchers to contribute to the field of bioinformatics as well as further the understanding of cellular dynamics.

We also intend to increase the depth of the data available for each cell and time-lapse image. One such approach would be to attempt to predict the movement of T cells based on CRAC channel information as well as surrounding T cells. The information gained from these two unique datasets, as well as future datasets, will be used to develop a comprehensive cell tracking algorithm that can be used for any cell type. The creation of an accurate system to model intercellular and intracellular dynamics based on a multitude of factors including external stimuli, surrounding cell types, and overall cellular population, can increase the speed at which different treatments can be evaluated, and reveal previously unknown interactions. Furthermore the creation of a robust in-silico model can increase the effectiveness of in-vivo and in-vitro cell tests by revealing potentially promising tests as well as illuminating possibly unwanted interference.

Additionally it should be noted that GCamp6f exhibits spontaneous fluorescence in the presence of any Ca$^{2+}$ signals inside the cell, therefor it is conceivable that the Ca$^{2+}$ increase detected in these images may be due to other Ca$^{2+}$ channels that may also be present in the cells. Thus the imaging protocol is not solely limited to detection of Ca$^{2+}$ via the CRAC channel, and the T-Time database approach may be widely applied to studies of numerous Ca$^{2+}$ influx mechanisms in the future.

In the long term we envision T-Time serving as an infrastructure for crowd sourcing data used to study cellular dynamics. Therefore the option to upload raw image files and retrieve images enhanced by the T-Time image enhancement algorithm will also be developed, along with the option to upload time series of cellular images and retrieve a tracked image set with corresponding metadata. The addition of these features will increase the ease with which live-cell imaging experiments can take place, while also increasing the amount of openly available live-cell imaging data.

## Conclusion

In pursuit of a more detailed understanding of the cellular landscape, the T-Time repository provides measures of morphology, locomotion, intensity of activation, and difference in intensity of phases. These features can be evaluated in different contexts in order to foster a more complete understanding of T cells. The data from each individual cell is available as a time-series of features, each of which provides a visual assessment on a frame-by-frame basis. Viewing of the tracked images, as well as untracked enhanced images, allows for independent verification of the tracking information captured by our algorithm. In order to fully understand the complex factors that influence cell activity, it is necessary to have a large amount of data readily available to the scientific community. As the understanding of cellular biology and the march towards personalized medicine accelerates, so does the need for a large repository of cellular images to distinguish differences among cell types. The T-Time repository provides an entry point for the creation of such a database.

## Abbreviations

BSA: bovine serum albumin
CRAC: Ca$^{2+}$ release-activated Ca$^{2+}$
GB: gigabyte
GECI: genetically encoded calcium indicator
ICAM-1: intercellular adhesion molecule-1
KB: kilobyte
MLAT: machine learning and assistive technology
PBS: phosphate buffered saline
SCID: severe combined immunodeficiency
SSDs: solid-state drives
.tif: tagged image format
